# Comparison of effects and safety in providing controlled hypotension during surgery between dexmedetomidine and magnesium sulphate: A meta-analysis of randomized controlled trials

**DOI:** 10.1371/journal.pone.0227410

**Published:** 2020-01-08

**Authors:** Bingchen Lang, Lingli Zhang, Yunzhu Lin, Wensheng Zhang, Feng-shan Li, Shouming Chen

**Affiliations:** 1 Department of Pharmacy, West China Second University Hospital, Sichuan University, Chengdu, People’s Republic of China; 2 Key Laboratory of Birth Defects and Related Diseases of Women and Children, Sichuan University, Ministry of Education, Chengdu, People’s Republic of China; 3 Evidence-Based Pharmacy Center, West China Second University Hospital, Sichuan University, Chengdu, People’s Republic of China; 4 Department of Anesthesiology, Laboratory of Anesthesia and Critical Care Medicine, Translational Neuroscience Center, West China Hospital, Sichuan University, Chengdu, People’s Republic of China; 5 Department of Anesthesiology, West China Second University Hospital, Sichuan University, Chengdu, People’s Republic of China; Cleveland Clinic, UNITED STATES

## Abstract

**Background:**

Effectiveness of controlled hypotension has been proven in alleviating intraoperative bleeding. Many recent studies emphasized the efficacy of dexmedetomidine and magnesium in providing controlled hypotension during various surgeries. The present meta-analysis of randomized controlled trials (RCTs) was performed to evaluate comprehensively the effects and safety of these two medications.

**Methods:**

Literature search was performed in four databases from inception to April 2019. All RCTs that used dexmedetomidine and magnesium as hypotensive agents were enrolled. The outcomes contained bleeding condition of surgical site, hemodynamic parameters, duration of surgeries, number of patients requiring opioid/analgesia administration, recovery period, and adverse events emerged during surgeries.

**Results:**

Ten studies with 663 patients met with our inclusion criteria. The results indicated that both bleeding score and values of mean arterial pressure (MAP) and heart rate (HR) were significantly lower in patients receiving dexmedetomidine (SMD 1.65 with 95% CI [0.90,2.41], *P*<0.00001) compared to the patients receiving magnesium. The effect in decreasing the necessity of using opioid/analgesia was affirmative in dexmedetomidine group (29.13% with magnesium vs 10.78% with dexmedetomidine), and the condition was more favorable in magnesium group in reducing recovery period (SMD -1.98 with 95% CI [-4.27,0.30], *P* = 0.09). Compared with magnesium, using of dexmedetomidine was associated with higher incidence of bradycardia but lower incidence of nausea and vomiting.

**Conclusion:**

Compared with magnesium, dexmedetomidine is more effective to provide promising surgical field condition, favorable controlled hypotension, and less necessity of opioid or analgesia administration. But long recovery period and high-probability bradycardia should be deliberated.

## Introduction

Intraoperative bleeding brought quite a crop of adverse effects including poor visibility of surgical site, increasing transfusion related reactions, and high rates of various complications [1]. As one approach performed in operations, intraoperative controlled hypotension has been proven effective in reducing blood loss, decreasing duration of surgery, and improving visibility of surgical sites [2,3]. Owing to the effects of decreasing arterial blood pressure, the introduction of controlled hypotensive anesthesia could also reduce the risks from angiorrhexis. It is desirable for various head and neck surgery including middle ear surgery, endoscopic procedures, neck dissection, and different plastic surgeries.

Miscellaneous medications can be used to induce hypotension during surgery including alpha2-adrenoreceptor agonists, magnesium sulfate, beta blockers, calcium channel blockers, sodium nitroprusside and many inhalation anesthetics. Therefore, to explore an ideal agent in providing controlled hypotension is a crucial issue. For anesthesiologists, the facility of administration, speed of onset and elimination, and the adverse effects of the agent would be the main concerns [4].

As one highly selective α2-adrenoceptor agonist (selectivity ratio for α2-adrenoceptor: α1-adrenoceptor is 1600:1) with sedative and analgesic characteristics, dexmedetomidine decreases the blood pressure owing to a decrease in norepinephrine and epinephrine plasma levels [5–7]. And rapid elimination and distribution make it an ideal option for intravenous infusion. Magnesium sulfate, one of non-competitive N-Methyl-D-Aspartate (NMDA) receptor antagonist, is employed as the hypotensive agent in diverse surgical procedures for several years [8]. On account of inhibition of the norepinephrine release by blocking N-type and partially L-type calcium channels, administration of magnesium can be served as one promising strategy for inducing controlled hypotension [9].

According to several recent studies, there has been a surge of interest in evaluating the effectiveness between dexmedetomidine and magnesium sulphate in ameliorating multiple indexes including hemodynamic parameters, bleeding score, vision of surgical site, duration of surgery, and the amount or frequency of opioid using. However, to our knowledge, no relevant study have been established to analyze systematically the effects of these two medications during different surgical procedures. Therefore, the objective of the present study was to provide a comprehensive meta-analysis from the existing evidences.

## Materials and methods

This present meta-analysis was performed according to the recommendations in the Preferred Reporting Items for Systematic Reviews and Meta Analyses (PRISMA) statement [10] and the guidelines described in the *Cochrane Handbook*.

### Search strategy

Two independent reviewers (BL and FL) performed the literature search. The databases including PubMed, Embase, Cochrane Library and China National Knowledge Infrastructure (CNKI) were searched systematically.

“Dexmedetomidine*”, “magnesium*”, “Precedex*”, “DEX” were combined in the search for relevant studies (Supporting information S1 File. Search strategy). The search was restricted to human studies, and the language of publications was not restricted. The last literature search was performed on April 21, 2019

### Inclusion and exclusion criteria

#### Participants

Adult patients with American Society of Anesthesiologists (ASA) physical status I-III of either sex who underwent general anesthesia and received magnesium sulphate or dexmedetomidine as hypotensive agent were included.

#### Interventions and comparisons

The intervention was administration of dexmedetomidine; the control group was given magnesium sulphate.

#### Outcome measurements

Given that controlled hypotension was served as one auspicious strategy for improving surgical condition and avoiding the occurrence of surgical bleeding, the primary outcome in present study was the bleeding condition at the surgical area which was evaluated by the 6-point scale [11] (0 = no bleeding; 1 = minor bleeding, no aspiration required; 2 = minor bleeding, aspiration required; 3 = minor bleeding, frequent aspiration required; 4 = moderate bleeding, visible only with the aspiration; and 5 = severe bleeding, continuous aspiration required, very hard to perform surgery).

The secondary outcomes contained hemodynamic profiles (Mean arterial pressure, MAP; Heart rate, HR), duration of surgery, number of patients requiring opioid/analgesia administration, and recovery profiles of patients (The time required to reach Aldrete score ≥9) [12]. The safety profiles of two medications including the incidence of participants who experienced any adverse events were also reviewed and analyzed systematically.

#### Studies

Randomized controlled trials (RCTs) with no language limitations were included. Studies were excluded if the data were from case reports, reviews, protocols or animal studies.

### Literature screening and data extraction

Two authors (BL and FL) performed the literature searching and information extraction independently, and then they cross-checked with each other. Full texts were checked when information from titles and abstracts could not be ascertained. According to the type of surgery, the general characteristics and information of selected studies were collected in our designed table (Table 1). Any disagreements were resolved by consensus through the discussion of all authors.

**Table 1:** The general characteristics of the enrolled studies.

**Type of surgery: head and neck surgery**										
**Study baseline** **characteristics**	Aboushanab *et al*., 2011 [14]		Akkaya *et al*., 2014 [15]		Bayram *et al*., 2015 [16]		Modir *et al*., 2018 [17]		Rokhtabnak *et al*., 2017 [18]	
**Surgery**	middle ear surgery		endoscopic sinus surgery		endoscopic sinus surgery		endoscopic sinus surgery/ tympanomastoidectomy		rhinoplasty	
**Treatment arm** **IV dosage**	Magnesium sulphate (n = 44)	Dexmedetomidine (n = 44)	Magnesium sulphate (n = 30)	Dexmedetomidine (n = 30)	Magnesium sulphate (n = 30)	Dexmedetomidine (n = 30)	Magnesium sulphate (n = 35)	Dexmedetomidine (n = 35)	Magnesium sulphate (n = 29)	Dexmedetomidine (n = 28)
	50 mg/kg followed by 15 mg/kg/h	1 μg/kg followed by 0.4–0.8 ug/kg/h	50 mg/kg followed by 15 mg/kg/h	1 μg/kg followed by 0.6 ug/kg/h	40 mg/kg followed by 10–15 mg/kg/h	1 μg/kg followed by 0.5–1 ug/kg/h	40 mg/kg followed by 10 mg/kg/h	1 μg/kg followed by 0.4 ug/kg/h	40 mg/kg followed by 10–15 mg/kg/h	1 μg/kg followed by 0.4–0.6 ug/kg/h
**ASA-physical status**	I-II	I-II	I-II	I-II	I-II	I-II	I-II	I-II	I	I
**Age, years**	32.4±7.3	34.1±6.4	42.9±15.1	42.5±16.1	39.5±11.3	45.1±11.1	33.4±6.27		25.6±7.1	32.4±7.3
**Gender (F/M)**	18/26	20/24	12/18	8/22	19/11	22/8	N/A		25/4	21/7
**Hemodynamic Parameters**	No significant differences neither in MAP nor in HR		MAP was lower in Group DEX at the 35^th^ and 65^th^ min; HR was lower in Group DEX at the 35^th^, 40^th^, and 45^th^ min.		MAP and HR were lower in Group DEX for all measurements except the initial stage.		MAP and HR were lower in Group DEX for all measurements.		No significant differences neither in MAP nor in HR	
**Bleeding at surgical area, Mean ± SD/ Median(Range)**	2.2 (1–4)	2.4 (1–4)	2.45±0.70	1.59±0.84	2.10±1.03	1.20±0.61	2.06±0.64	1.11±0.40	3.69±1.07	2.32±1.02
**Duration of surgery, min**	116±18	118±17	50.2±18.6	51.0±14.8	61.5±17.3	55.9±18.3	126.34±6.2	125.26±6.2	133.2x45.6	144±40.8
**Patients requiring opioid / analgesia administration**	10 (22.7%)	8 (18.2%)	N/A	N/A	7 (23.3%)	1 (3.3%)	N/A	N/A	13 (44.8%)	2 (7.1%)
**Time till reaching Aldrete score ≥9, min**	38.2±5.2	60.7±6.8	N/A	N/A	14.8±3.0	11.8±2.5	30.83±4.8	46.03±5.3	15±2.89	22.53±3.56
**Type of surgery: Clinical Spine Surgery**							**Other types of surgery**			
**Study baseline** **characteristics**	Oommen *et al*., 2018 [19]		Radwan *et al*., 2017 [20]		Srivastava *et al*. 2016 [21]		Soliman *et al*. 2017 [22]		Zarif *et al*. 2016 [23]	
**Surgery**	elective lumbar spinal fusion surgery		elective anterior corpectomy and cervical fixation		elective spine surgery		elective surgery of pituitary adenoma		Laparoscopic colorectal resection	
**Treatment arm** **IV dosage**	Magnesium sulphate (n = 21)	Dexmedetomidine (n = 21)	Magnesium sulphate (n = 20)	Dexmedetomidine (n = 20)	Magnesium sulphate (n = 30)	Dexmedetomidine (n = 30)	Magnesium sulphate (n = 76)	Dexmedetomidine (n = 76)	Magnesium sulphate (n = 17)	Dexmedetomidine (n = 17)
	30 mg/kg followed by 10 mg/kg/h	1 μg/kg followed by 0.4 ug/kg/h	30 mg/kg followed by 10 mg/kg/h	1 μg/kg followed by 0.5 ug/kg/h	50 mg/kg followed by 15 mg/kg/h	1 μg/kg followed by 0.5 ug/kg/h	50 mg/kg followed by 15 mg/kg/h	1 μg/kg followed by 0.5 ug/kg/h	2g followed by 15 mg/kg/h	1 μg/kg followed by 0.4 ug/kg/h
**ASA-physical status**	I-II	I-II	I-II	I-II	I-II	I-II	I-II	I-II	I-III	I-III
**Age, years**	31–60	19–60	32.05±5.79	32.25±6.07	48.30±7.70	45.93±9.19	44.02±10.14	43.20±10.93	60.3±7.3	60.9±7.1
**Gender (F/M)**	8/13	10/11	8/12	9/11	13/17	12/18	40/36	36/40	4/13	5/12
**Hemodynamic Parameters**	MAP were lower in Group MgSO_4_ for all measurements; HR was lower in Group DEX from the 15^th^ min		MAP was lower in Group DEX at time of intubation; HR was lower in Group DEX at time of intubation		MAP was lower in Group DEX from the 60^th^ min; HR were lower in Group DEX for all measurements		MAP and HR were lower in Group DEX for all measurements.		No significant differences neither in MAP nor in HR	
**Duration of surgery, min**	N/A	N/A	N/A	N/A	144.6±28.8	157.2±35.4	195.45±27.66	192.74±30.55	128.8±23.8	129.4±12.4
**Bleeding at surgical area (6-point scale), Mean ± SD / Median(Range)**	N/A	N/A	N/A	N/A	N/A	N/A	3.05±0.65	1.36±0.48	N/A	N/A
**Blood loss during surgery, ml**	516±8.03	499±5.34	N/A	N/A	N/A	N/A	299.47±77.28	157.43±48.79	295.9±97.2	281.5±50.3
**Time till reaching Aldrete score ≥9, min**	N/A	N/A	N/A	N/A	N/A	N/A	N/A	N/A	24.1±7.5	23.2±6.4

Abbreviation: N/A, not applicable, *DEX* Dexmedetomidine, *MAP* Mean arterial pressure, *HR* Heart rate.

### Quality assessment

On the basis of Cochrane Collaboration tool for assessing risk of bias in randomized trials, two authors (BL and FL) independently evaluated the methodological quality involving seven aspects: random sequence generation, allocation concealment, blinding of participants and personnel, blinding of outcome assessment, incomplete outcome data, selective reporting and other bias [13].

### Statistics analysis

Statistical analyses were employed by using STATA/SE 12.0 statistical package (StataCorp LP, College Station, TX, USA). The risk ratio (RR) with 95% confidence interval (CI) and the Mantel–Haenszel method (fixed or random models) were used to analyze dichotomous data. For continuous data, standardized mean difference (SMD) was chosen for the estimation. The *I*-squared (*I*^2^) test was chosen to weigh the impact of heterogeneity on the results. As described by the Cochrane review guidelines, the random-effects model was chosen when severe heterogeneity was present at *I*^2^>50%; otherwise, the fixed-effects model was applied. Additionally, we performed the sensitivity analysis by deleting each study individually to assess the quality and consistency of the results. Publication bias was evaluated by using Begg’s test and Egger’s test. A *P* value less than 0.05 was considered statistically significant.

## Results

### Literature search results

**Fig 1:**
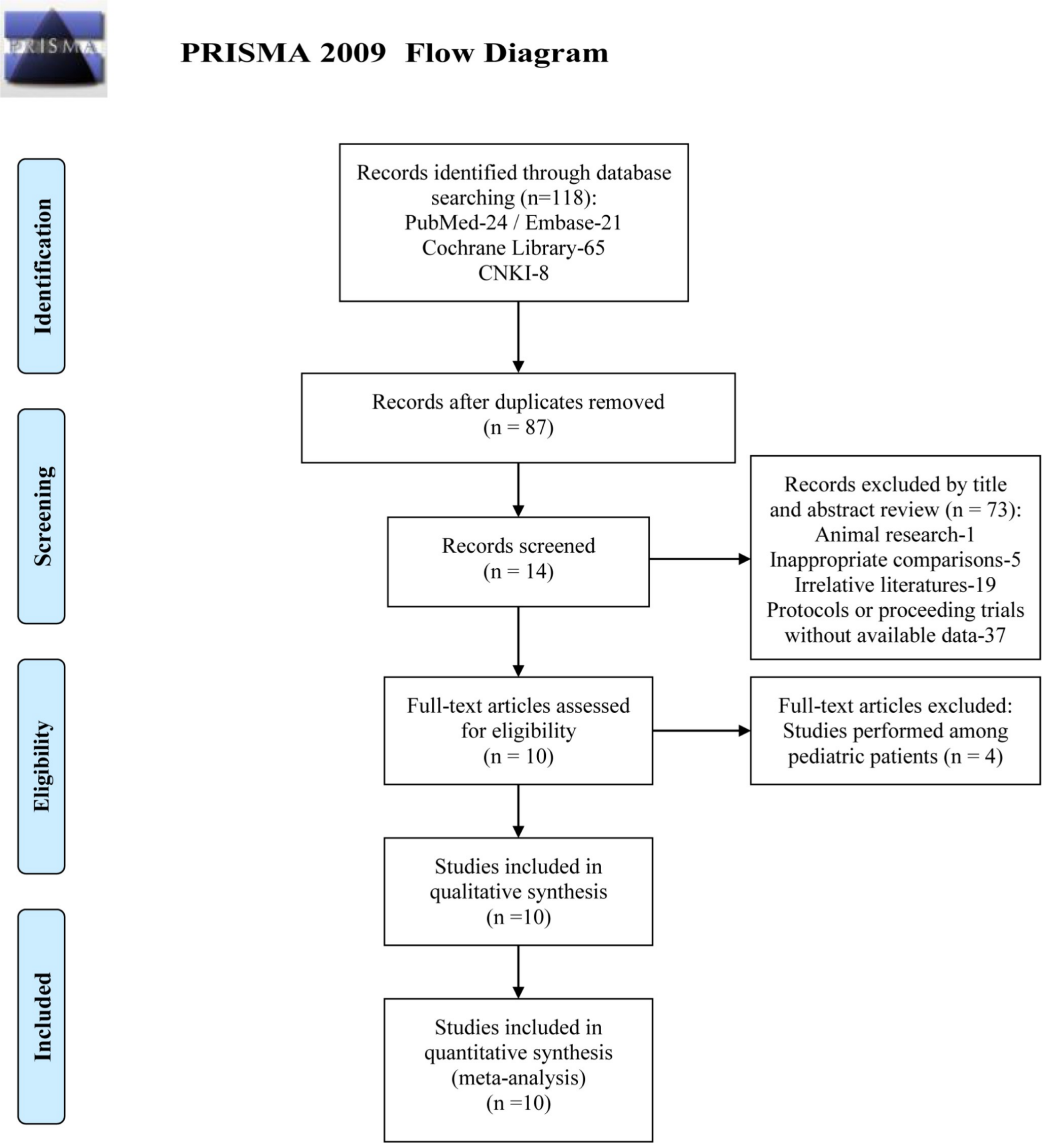
Flow chart of literature screening and the selection process. Abbreviation: CNKI, China National Knowledge Infrastructure.

In 118 identified articles, 31 were excluded after duplicate removing, and 73 were excluded after the title and abstract review. In these 73 excluded items, 1 was animal research, 5 were inappropriate comparisons (e.g. the comparison between dexmedetomidine and midazolam/propofol), 11 were uncorrelated outcomes (e.g. the efficacy of two medications in reducing fentanyl induced cough, the analgesia efficacy of two medications as adjuvants in local anesthetic), 19 were irrelative case reports, meetings, commentaries and reviews, 37 were protocols or trials which were being processed without data available for analyzing. And then 4 studies were excluded by full-text review for they were performed in pediatric patients. At last, 10 RCTs were enrolled in further meta-analysis [14–23]. The identification procedure of these eligible articles is described in Fig 1. The enrolled studies were published from 2011 to 2018, and all of them were published in English.

### Basic characteristics of enrolled studies

A total of 663 patients were randomized to receive two drugs. In 10 included RCTs, 5 were researches about head and neck surgery [14–18], 3 were researches about spinal surgery [19–21], and the rest two trials were researches about pituitary adenoma surgery [22] and laparoscopic colorectal resection surgery [23]. All enrolled studies reported the hemodynamic parameters (MAP, HR), and 6 studies reported the evaluation of surgical field bleeding by using 6-point scale [14–18,22]. Duration of surgery was reported in 8 studies [14–18,21–23], and the number of patients requiring opioid/analgesia administration was reported in 3 studies [14,16,18]. The recovery profile of patients was measured by the time till reaching Aldrete score ≥9 in 5 studies [14,16–18,23]. The general characteristics and information of enrolled studies are described in Table 1.

### Quality assessment

**Fig 2:**
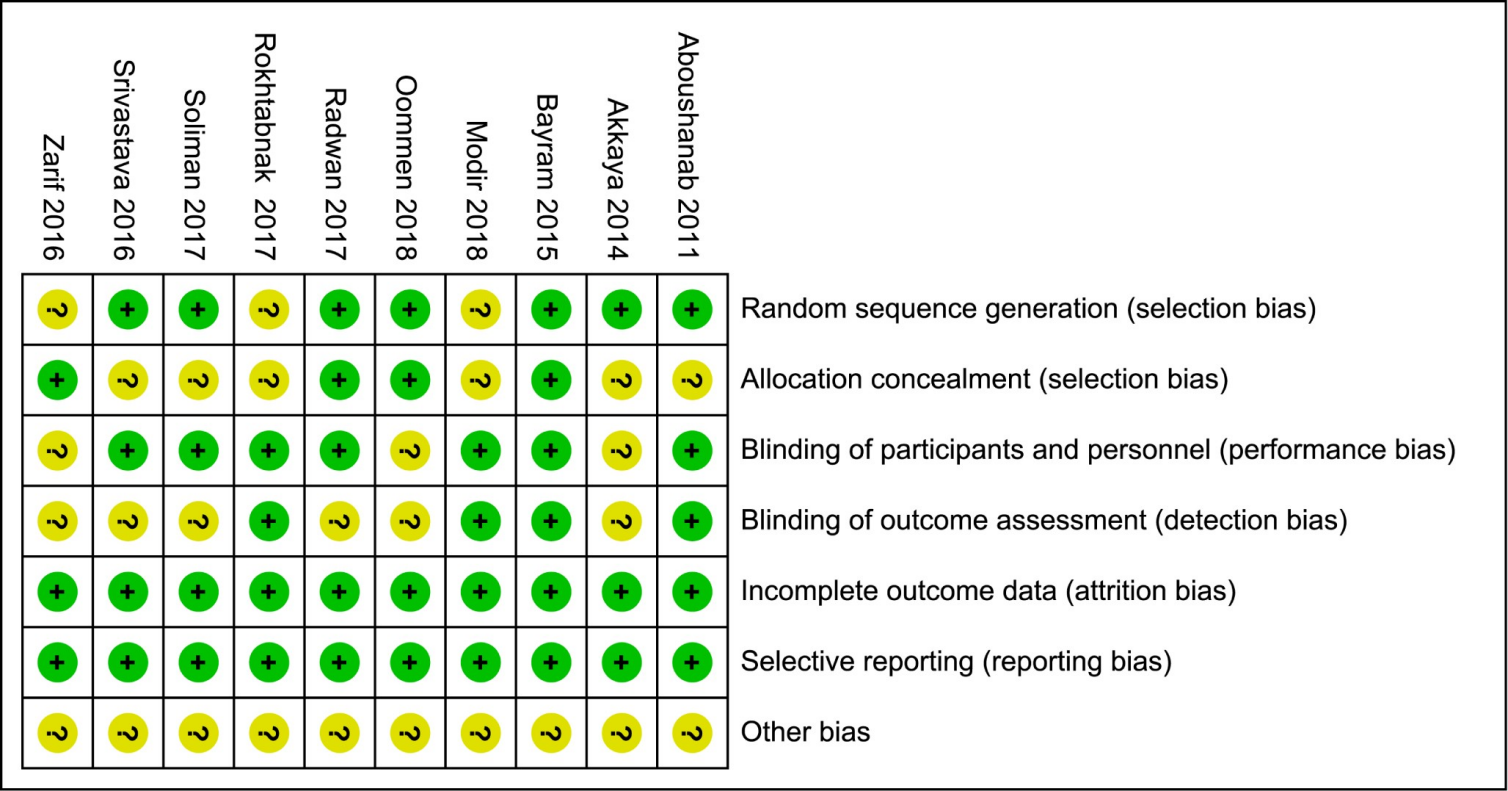
Risk of bias assessment of included studies. Notes: Green + dot, low risk of bias; yellow ? dot, unclear risk of bias; red—dot, high risk of bias.

The mentioned-above seven aspects including random sequence generation, allocation concealment, blinding of participants and personnel, blinding of outcome assessment, incomplete outcome data, selective reporting and other bias of selected RCTs were evaluated according to the Cochrane Collaboration tool. Seven studies used an adequate method of random sequence generation [14–16,19,20,21,22], and four studies [16,19,20,23] employed allocation concealment by using the opaque, sealed envelopes. Seven studies mentioned the blinding procedure of participants and personnel [14,16–18,21,22] and the blinding procedure of outcome assessment was performed appropriately in four studies [14,16–18]. The profile of risk of bias assessment tool is shown in Fig 2.

### Assessment of the bleeding condition of surgical field

**Fig 3:**
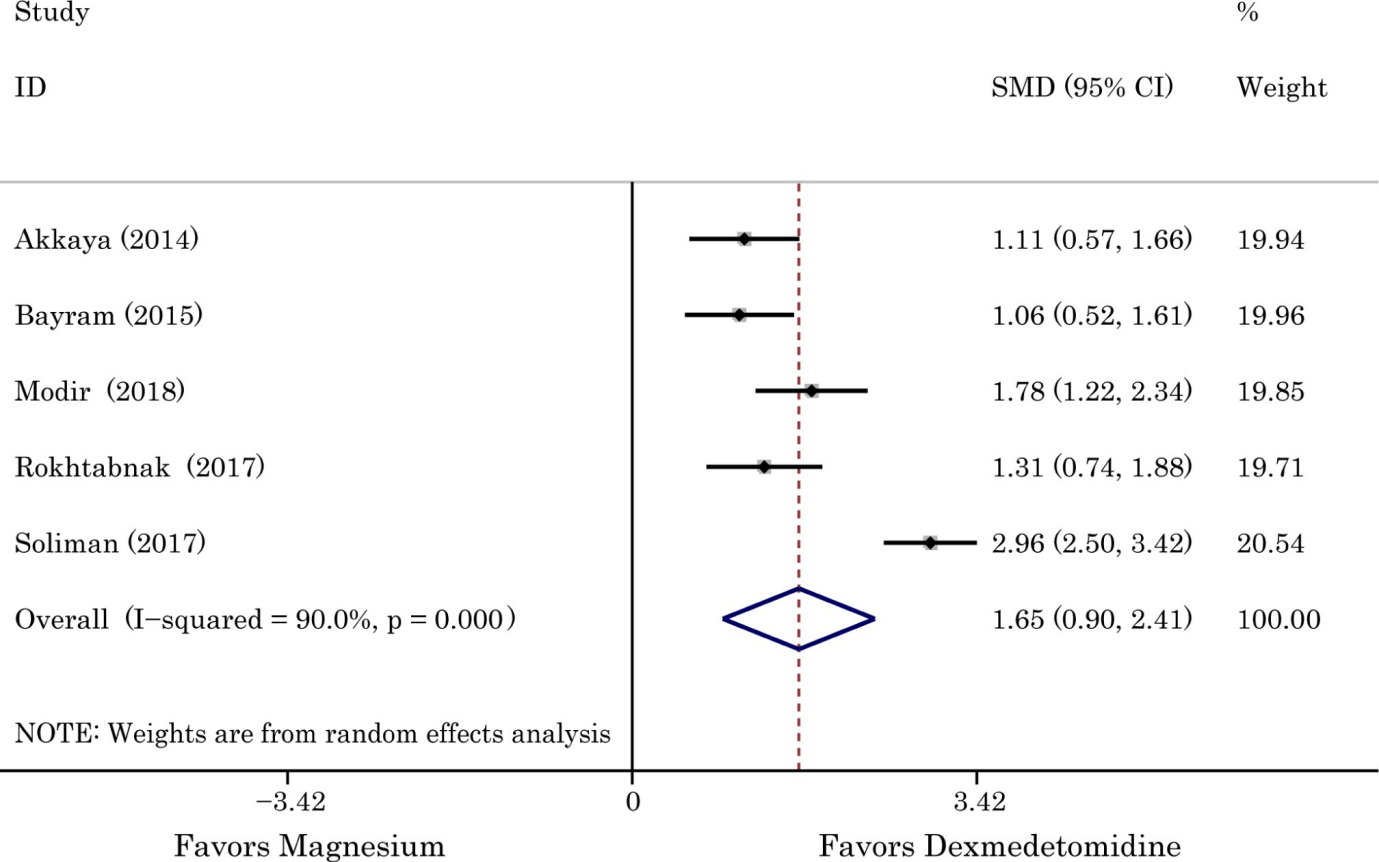
The effects of dexmedetomidine and magnesium in bleeding and visibility of surgical field. Abbreviation: *SMD* standardized mean difference, *CI* confidence interval.

**Fig 4:**
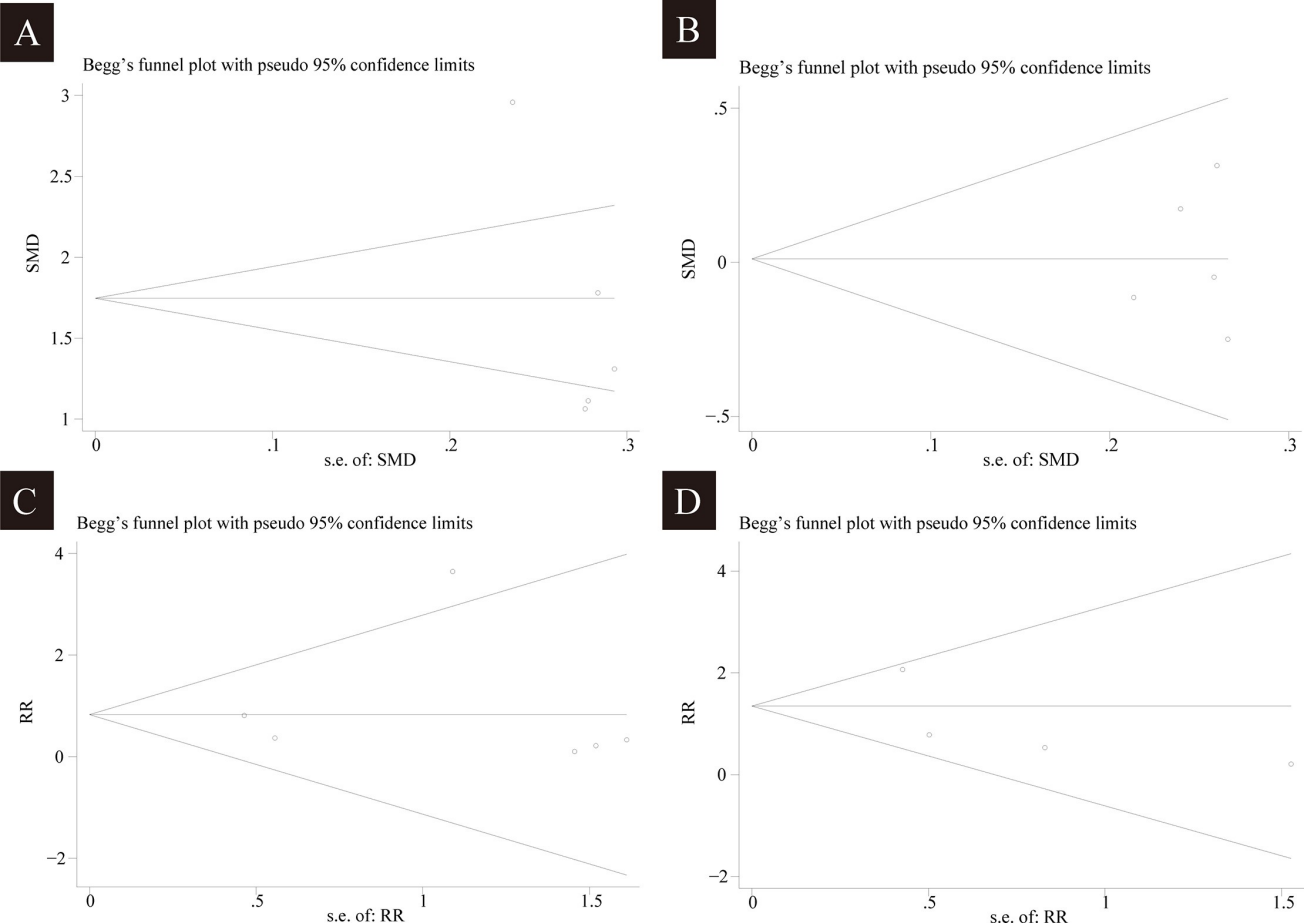
Funnel plots of effect estimates for various clinical outcomes. Notes: Dexmedetomidine vs Magnesium (A) the bleeding condition of surgical field: Egger’s tests, *P* = 0.047; (B) duration of surgery: Egger’s test, *P* = 0.812; (C) Bradycardia Incidence: *P* = 0.764; (D) Hypotension Incidence: *P* = 0.365. Abbreviations: *RR*, risk ratio; *SE*, standard error.

The bleeding condition of the surgical site was graded on a 6-point scale which described the intraoperative bleeding and visibility of surgical field. Given that the scores in Aboushanab study (Aboushanab et al., 2011) [14] were calculated in median (range), a total of five studies involving 399 patients were included, and 200 of them were received the magnesium sulphate. Considering the statistical heterogeneity exists among the study results (*I*^*2*^ = 90%), the random-effects model was chosen. According to the results of data combination, compared to administration of magnesium, bleeding score was significantly decreased in patients receiving the infusion of dexmedetomidine (SMD 1.65 with 95% CI [0.90,2.41], *P<*0.00001, *I*^2^ = 90%). The *I*^2^ of 90% demonstrated the existing heterogeneity which was attributable to the Soliman study (Soliman *et al*., 2017) [22]. After removing this study, heterogeneity was resolved (*I*^2^ = 26%) with the unchanged summary estimate in essence (SMD 1.31 with 95% CI [0.99,1.63], *P*<0.00001, *I*^2^ = 25%). The result is shown in Fig 3. The results of Egger’s (*P* = 0.047) tests indicated publication bias was existed (Fig 4A). Hence, the Duval’s trim and fill method was performed to estimate and adjust for the number and outcomes of missing studies [24]. And results from sensitivity analyses of trim and fill method (no new studies added) exhibited that the result was reliable.

### Effects on hemodynamic parameters (MAP and HR)

**Fig 5:**
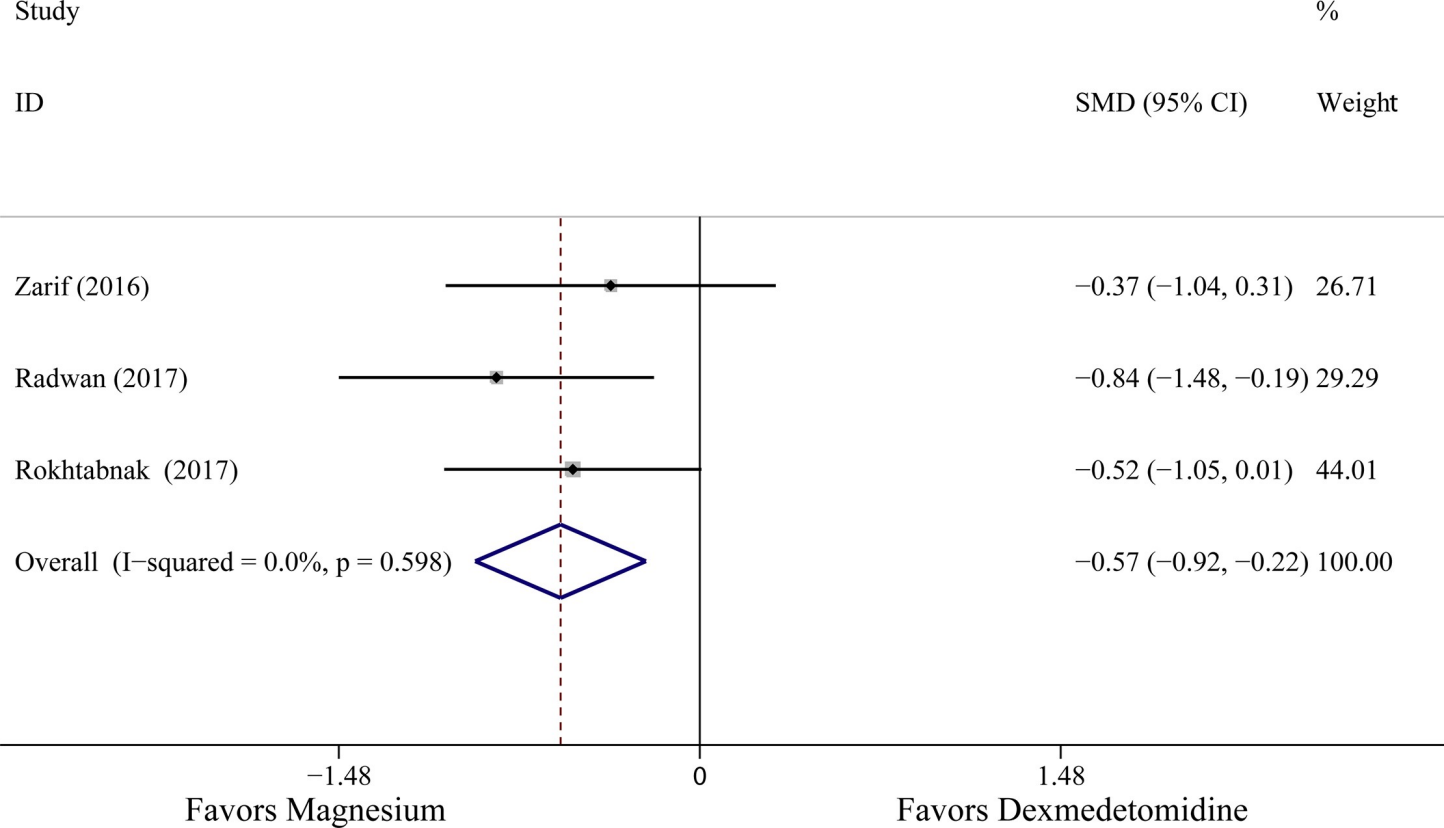
The effects of dexmedetomidine and magnesium on mean arterial pressure. Abbreviation: SMD standardized mean difference, CI confidence interval.

**Fig 6:**
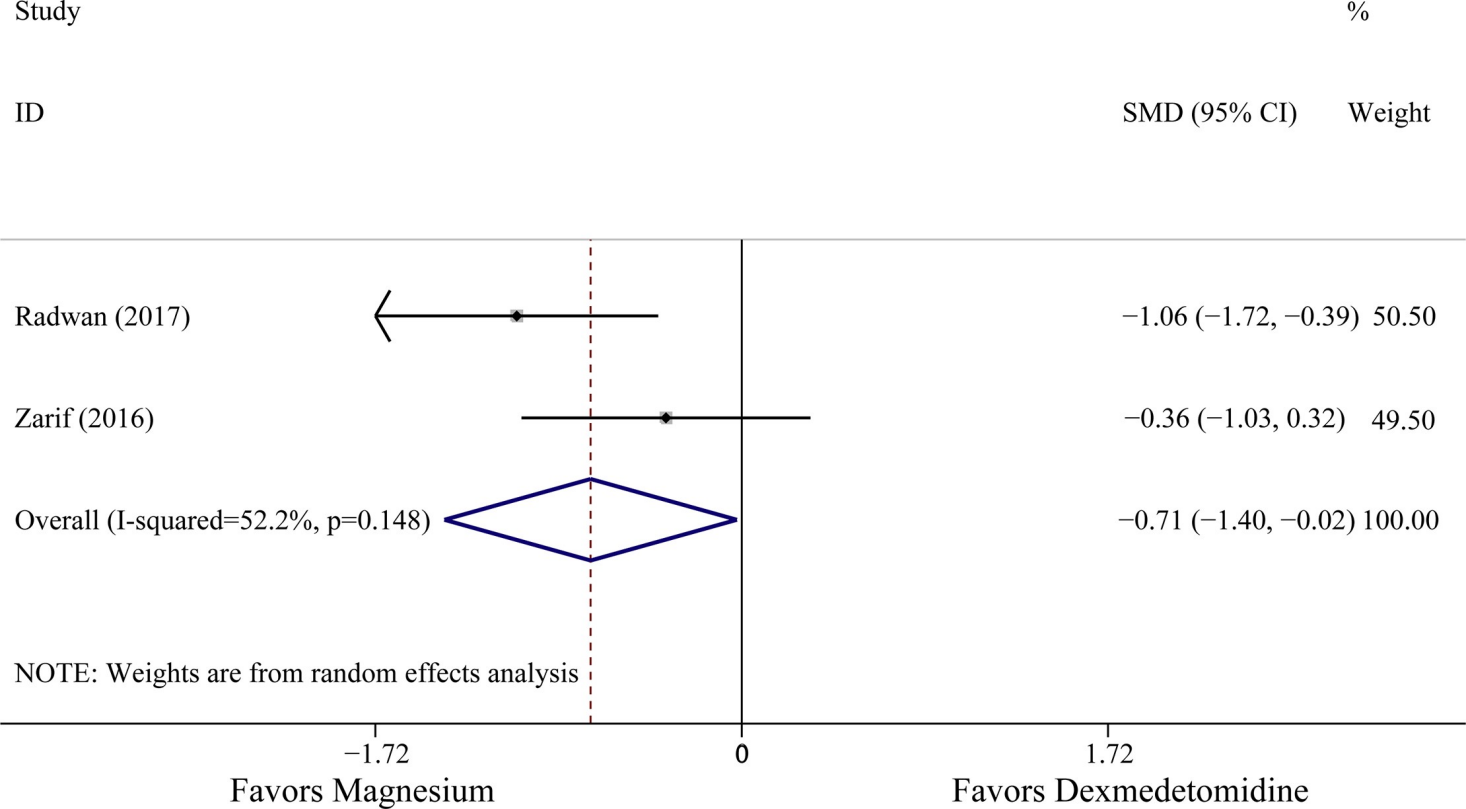
The effects of dexmedetomidine and magnesium on heart rate. Abbreviation: SMD standardized mean difference, CI confidence interval.

All ten studies described the effects of two medications in patients’ hemodynamic parameters. The level of MAP observed in six studies was lower in patients receiving dexmedetomidine [15–17, 20–22], and three studies [14,18,23] exhibited that the differences between two groups was not significant, only one study [19] indicated that the MAP of patients receiving magnesium sulphate was lower. In addition, according to the original data from seven studies [15–17,19–22], compare with those receiving magnesium, the patients in dexmedetomidine group exhibited lower HR values. In fact, the haemodynamic parameters were always measured repeatedly during drug administration or presented in chart, thus, the minimum values of the haemodynamic responses (HR, MAP) were extracted if the data were available in original text. Three RCTs involving 131 patients and two RCTs involving 74 patients were included in analysis of MAP and HR, the results indicated that the administration of dexmedetomidine was associated with the lower MAP and HR minimum values compared to magnesium (MAP: SMD -0.57 with 95% CI [-0.92,-0.22], *P* = 0.001, *I*^2^ = 0%; HR: SMD -0.71 with 95% CI [-1.40,-0.02], *P* = 0.003, *I*^2^ = 52.2%). The results are shown in Fig 5 and Fig 6.

### Duration of surgery

**Fig 7:**
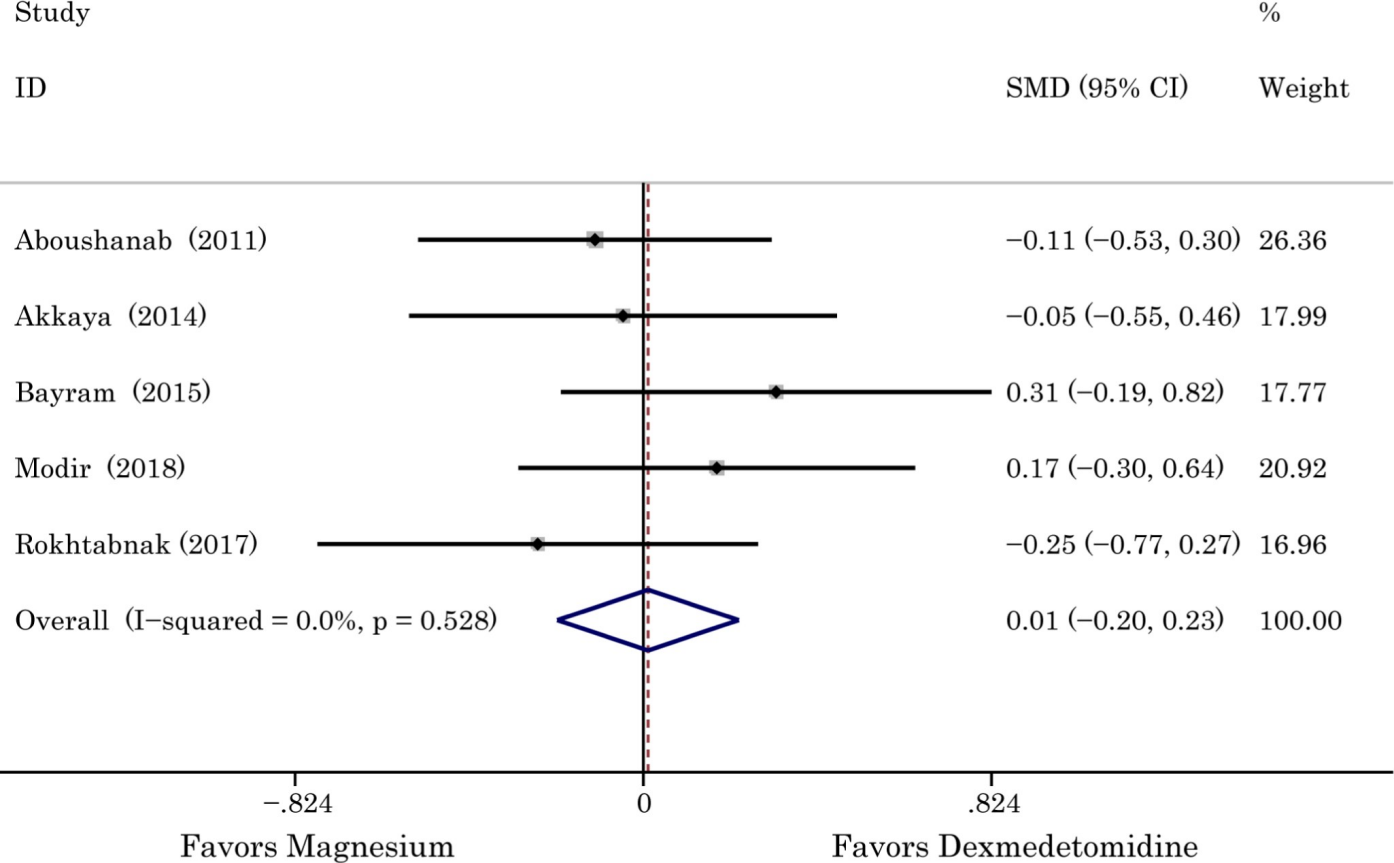
The effects of dexmedetomidine and magnesium in duration of surgery. Abbreviation: *SMD* standardized mean difference, *CI* confidence interval.

According to original data from three studies (one was the study of spine surgery, one was the study of pituitary adenoma surgery, and one was the study of laparoscopic colorectal resection surgery), the difference between magnesium sulphate group and dexmedetomidine group in length of operation time was not significant. Our analysis enrolled five studies (head and neck surgery) involving 335 participants, 168 of them were given magnesium sulphate. The *I*^2^ of 0 demonstrated that there was no existence of substantial heterogeneity, thereby the fixed-effect model was used. The results indicated that no significant difference was found between the two groups in duration of surgery (SMD 0.01 with 95% CI [-0.20,0.23], *P =* 0.92, *I*^2^ = 0%). The result is shown in Fig 7. By using both Begg’s (*P*>1.000) and Egger’s (*P* = 0.812) tests, publication bias was found in the analysis (Fig 4B).

### Number of patients requiring opioid/analgesia administration

**Fig 8:**
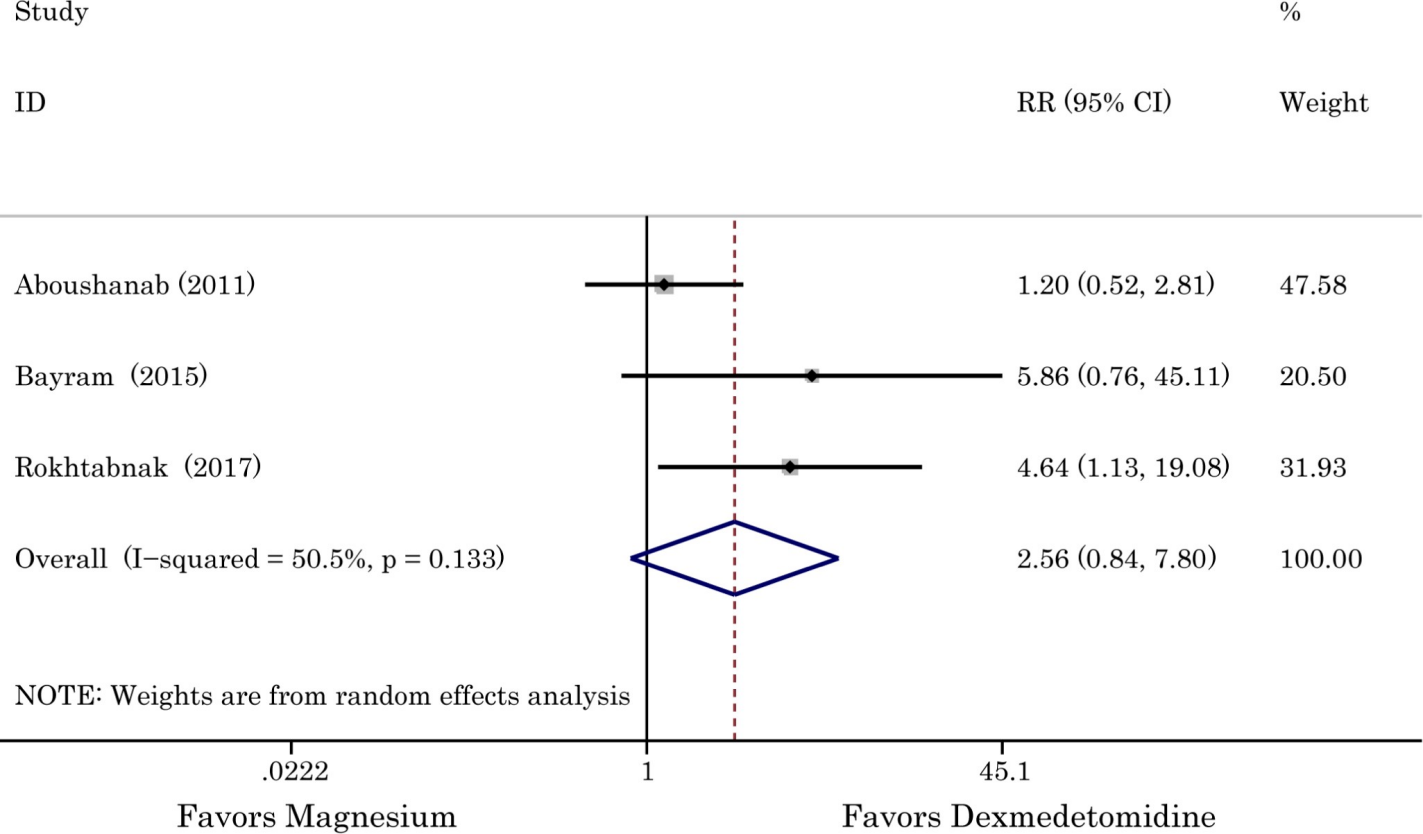
The effects of dexmedetomidine and magnesium in decreasing the necessity of using opioid or analgesia. Abbreviation: *RR* risk ratio, *CI* confidence interval.

A total of three RCTs (head and neck surgery) involving 205 patients were analyzed, 103 of them were administrated by magnesium sulphate. The *I*^2^ of 50.5% revealed that the substantial heterogeneity was existed, thus, the random-effects model was applied. Although the difference between two groups in number of patients requiring opioid/analgesia administration was not significant, the results indicated that the condition was more favorable in dexmedetomidine group (incidence of patients requiring opioid/analgesia: 29.13% with magnesium *vs* 10.78% with dexmedetomidine, RR with 95% 2.56 [0.84, 7.80], *P* = 0.08, *I*^*2*^ = 50.5%). The result is shown in Fig 8.

### Recovery profiles

**Fig 9:**
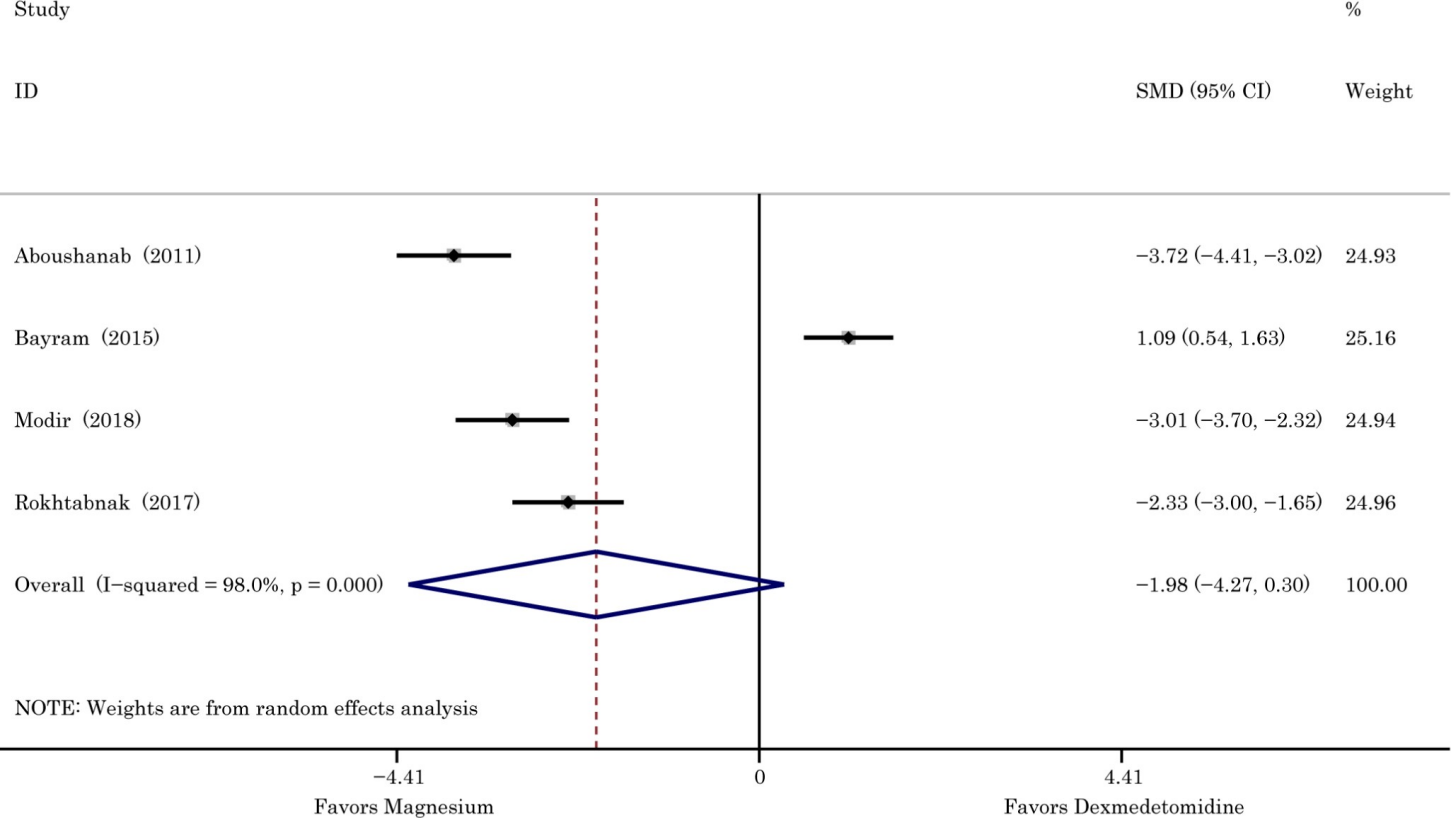
The effects of dexmedetomidine and magnesium in reducing recovery period. Abbreviation: *SMD* standardized mean difference, *CI* confidence interval.

The postoperative recovery profiles of patients were evaluated by the modified Aldrete score, and the time to reach score≥9 represents the recovery period [12]. The original data from Zarif study (Zarif *et al*., 2016) indicated that the difference between magnesium sulphate group and dexmedetomidine group in time till reaching Aldrete score≥9 was not significant. Analysis of this outcome included four studies about head and neck surgery, and 275 patients were involved. The *I*^*2*^ of 98% demonstrated the substantial heterogeneity existed, then the random-effects model was employed. The results of data combination indicated that the condition was more favorable in magnesium sulphate group in time needed to reach Aldrete score≥9 (SMD -1.98 with 95% CI [-4.27,0.30], *P =* 0.089, *I*^2^ = 98%). The *I*^2^ of 98% indicated substantial heterogeneity but the source could not be exactly attributed to a single study (Fig 9).

### The summary of adverse events

Adverse events were mentioned in 7 studies. Four major types of adverse events including nausea/vomiting, bradycardia, hypotension, and shivering were reported in 62 patients of magnesium sulphate group and in 59 patients of dexmedetomidine group respectively. According to the results, treatment with magnesium sulphate resulted in higher incidence of nausea/vomiting (14.07% with magnesium sulphate *vs* 5.22% with dexmedetomidine, RR = 2.39, with 95% CI [1.06, 5.36], *P =* 0.035, *I*^2^ = 0.0%) and lower incidence of bradycardia (5.73% with magnesium sulphate *vs* 11.54% with dexmedetomidine, RR = 0.55, with 95% CI [0.31, 0.98], *P =* 0.042, *I*^2^ = 16.9%) compared to dexmedetomidine. There were no significant differences between two groups in number of patients who experienced hypotension needed vasoactive intervention and in number of patients who experienced shivering. The analysis of adverse effects were described in Table 2. The results of Begg’s test (Bradycardia Incidence: *P*>1.000; Hypotension Incidence: *P*>1.000) and Egger’s test (Bradycardia Incidence: *P* = 0.764; Hypotension Incidence: *P* = 0.365) indicated that the publication bias was not existed (Fig 4C and Fig 4D).

**Table 2:** Adverse events for Magnesium sulphate versus Dexmedetomidine.

Adverse event	Number of studies [reference no.]	Number of pooled events/participants (Rate, %)		Total sample size	*I*^*2*^	Risk ratio (95% CI)	*P* value
		Magnesium sulphate	Dexmedetomidine				
**Nausea/Vomiting**	3 [16,18,22]	19/135 (14.07%)	7/134 (5.22%)	269	0.0%	2.39 [1.06, 5.36]	***0*.*035***
**Bradycardia**	**7** [14,16–18,20–22]	15/262 (5.73%)	30/260 (11.54%)	522	16.9%	0.55 [0.31, 0.98]	***0*.*042***
**Hypotension**	6 [14,16–18,21,22]	24/242 (9.92%)	21/240 (8.75%)	482	34.7%	1.11 [0.64, 1.93]	0.716
**Shivering**	2 [16,18]	4/59 (6.78%)	1/58 (1.72%)	117	0.0%	2.81 [0.46, 17.21]	0.263

## Discussion

As one effective approach, application of the controlled hypotension has been generalized in different surgical procedures to improve operative field visibility. Many different drugs have been proven effective in inducing hypotension and in ameliorating various intraoperative and postoperative parameters including quality of surgical site, duration of surgery, amount of opioid or analgesia using, and recovery period. The focus from recent studies moved on the efficacy assessment of NMDA receptor antagonist (such as magnesium sulphate) [8] and α2-adrenoceptor agonist (such as dexmedetomidine) [25]. Although many studies emphasized the ability of two above-mentioned drugs in providing controlled hypotension during operation, there were limited evidences to systematically review the efficacy and safety between the two medications. Clinicians and anesthesiologists may expect to identify the preferred option for clinical application.

We thereby conducted the first meta-analysis of the published RCTs to compare the effects and safety between the two medications in providing controlled hypotension during surgery by evaluating miscellaneous parameters including surgical vision quality, intraoperative and postoperative profiles, and the occurrence of adverse events. To make the existing evidences as comprehensive as possible, a thorough search including not only several international but also a Chinese database was performed by us. And the accessible studies we obtained were all published in English.

Ten studies enrolled in eventual quantitative synthesis covered different types of surgeries such as head and neck surgeries and spine surgeries, thus, the analyses classified by surgery types were performed in our study.

The investigation of dexmedetomidine and magnesium sulphate in improving the quality of surgical site indicated that the using of former one was associated with significantly lower 6-point bleeding score and better quality of surgical site. According to some relevant studies [26–28], decreasing the HR can be served as one strategy to provide a better operative field condition, and there was no need to decrease MAP to the risky low levels. In our present study, all included RCTs reported the effects of two drugs in hemodynamic parameters including MAP and HR. Compared with magnesium, the overall tendency about the effects of dexmedetomidine in producing lower value of both MAP and HR was observed. The results were consistent with the description about the outcomes on assessment of 6-point bleeding score.

The results about the effects of two medications in duration of surgical procedure demonstrated that the difference between two groups was not significant. In addition, the analysis from three studies related to head and neck surgery revealed that dexmedetomidine provided a favorable condition in decreasing the necessity of analgesia or opioids administration during operation rather than magnesium sulphate. The results strengthened the previous findings and illustrated the potential analgesic effects from alpha-2 agonist [29]

There is not only one approach for assessing objectively the progress of post-anaesthesia recovery. The scoring system proposed by Steward in 1975 [30], for instance, involved the evaluation of consciousness, airway and movement. The Post Anesthetic Recovery (PAR) Score system proposed by Aldrete et al. assessed the activity, consciousness, circulation, respiration and color, is used as mainstream in many countries [31,32], The recovery profiles of patients described by time till reaching Aldrete score ≥9 were reported in all five studies, and the results from four studies related to head and neck surgery indicated that the recovery condition was favorable in patients receiving magnesium sulphate. It may be resulted from the effect of magnesium in decreasing the anesthetic requirements [8] and the sedative effects of intravenous dexmedetomidine, and it was observed in three included studies that patients receiving dexmedetomidine needed longer time to reach modified Aldrete score 9. According to the summary of adverse events, more patients experienced nausea or vomiting in magnesium sulphate group. But bradycardia occurred more frequently in patients receiving dexmedetomidine, which may be resulted from its action on α2 agonist receptors [5]. The differences of hypotension (required vasoactive intervention) incidence and shivering incidence between two groups were not significant.

As far as we know, the present study is the first meta-analysis to compare the effects and safety between dexmedetomidine and magnesium sulfate in providing controlled hypotension. However, some limitations of the current study should be taken into account. Although the search strategy and the target databases were designed to perform the evidence screening as thorough as possible, both the number of RCTs met the eligibility criteria and the sample size of enrolled studies were limited. Additionally, different types of surgeries from the included studies led to absence or dissimilarity of the clinical data. Therefore, we performed our analyses by classifying the surgical types to avoid the heterogeneity. Overall, to strengthen the reliability of conclusion gathering from the clinical trials, more high-quality evidences with large sample size are required in future.

### Conclusion

According to the current evidences, compared with magnesium sulphate, dexmedetomidine is a more effective approach in producing controlled hypotension with favorable surgical field condition and low frequency of opioid/analgesia administration. Nevertheless, the using of dexmedetomidine associated with the long recovery period and increased risk of bradycardia. These are two main concerns need to be deliberated when selecting it as the hypotensive agent during surgical procedure. Furthermore, given that the existing evidences are limited, more high-quality studies with large sample size is necessary in future.

## Supporting information

S1 File Search strategy. (DOCX)

S2 File PRISMA checklist. (DOC)

## Abbreviations

RCTs: randomized controlled trials
MAP: mean arterial pressure
HR: heart rate
NMDA: receptor antagonist, N-Methyl-D-Aspartate receptor antagonist
PRISMA: Preferred Reporting Items for Systematic Reviews and Meta Analyses
ASA: American Society of Anesthesiologists
CI: confidence interval
SMD: standardized mean difference
