# Supplementary material for: Comparison of effects and safety in providing controlled hypotension during surgery between dexmedetomidine and magnesium sulphate: A meta-analysis of randomized controlled trials
Source: PLoS One. 2020 Jan 8;15(1):e0227410. doi: 10.1371/journal.pone.0227410 (PMC6949117; doi:10.1371/journal.pone.0227410)
Supplement: S1 File — (DOCX) [file pone.0227410.s001.docx]

2019.4.21

Pubmed

#1 (randomized controlled trial [pt] OR controlled clinical trial[pt] OR randomized[tiab] OR placebo[tiab] OR clinical trials as topic[mesh: noexp] OR randomly[tiab] OR trial[ti]) NOT (animals[mh] NOT humans[mh])

#2 magnesium*

#3 dexmedetomidine* OR Precedex* OR DEX

#1 AND #2 AND #3

| Search | Query | Items found |
| --- | --- | --- |
| [#5](https://www-ncbi-nlm-nih-gov.elibrary.einstein.yu.edu/pubmed/advanced) | Search ((((randomized controlled trial [pt] OR controlled clinical trial[pt] OR randomized[tiab] OR placebo[tiab] OR clinical trials as topic[mesh: noexp] OR randomly[tiab] OR trial[ti]) NOT (animals[mh] NOT humans[mh]))) AND magnesium*) AND (dexmedetomidine* OR Precedex* OR DEX) | [24](https://www-ncbi-nlm-nih-gov.elibrary.einstein.yu.edu/pubmed/?cmd=HistorySearch&querykey=5) |
| [#4](https://www-ncbi-nlm-nih-gov.elibrary.einstein.yu.edu/pubmed/advanced) | Search Remifentanil* OR Ultiva* | [4725](https://www-ncbi-nlm-nih-gov.elibrary.einstein.yu.edu/pubmed/?cmd=HistorySearch&querykey=4) |
| [#3](https://www-ncbi-nlm-nih-gov.elibrary.einstein.yu.edu/pubmed/advanced) | Search dexmedetomidine* OR Precedex* OR DEX | [13605](https://www-ncbi-nlm-nih-gov.elibrary.einstein.yu.edu/pubmed/?cmd=HistorySearch&querykey=3) |
| [#2](https://www-ncbi-nlm-nih-gov.elibrary.einstein.yu.edu/pubmed/advanced) | Search magnesium* | 104269 |
| [#1](https://www-ncbi-nlm-nih-gov.elibrary.einstein.yu.edu/pubmed/advanced) | Search (randomized controlled trial [pt] OR controlled clinical trial[pt] OR randomized[tiab] OR placebo[tiab] OR clinical trials as topic[mesh: noexp] OR randomly[tiab] OR trial[ti]) NOT (animals[mh] NOT humans[mh]) | 1121963 |

Embase

1 'randomised controlled trial'/exp

2 'magnesium'

3 'dexmedetomidine' OR 'Precedex' OR 'DEX'

4 1 and 2 and 3 (21)

| Search | Query | Items found |
| --- | --- | --- |
| [#4](https://www-ncbi-nlm-nih-gov.elibrary.einstein.yu.edu/pubmed/advanced) | #1 AND #2 AND #3 | 21 |
| [#3](https://www-ncbi-nlm-nih-gov.elibrary.einstein.yu.edu/pubmed/advanced) | Search 'dexmedetomidine' OR 'Precedex' OR 'DEX' | 22174 |
| [#2](https://www-ncbi-nlm-nih-gov.elibrary.einstein.yu.edu/pubmed/advanced) | Search 'magnesium' | 161401 |
| [#1](https://www-ncbi-nlm-nih-gov.elibrary.einstein.yu.edu/pubmed/advanced) | Search 'randomised controlled trial'/exp | 550636 |

Cochrane Library

#1 magnesium*

#2 dexmedetomidine* OR Precedex* OR DEX

#3 #1 AND #2 (65) (2 protocols, 6 reviews and 57 trials)

| Search | Query | Items found |
| --- | --- | --- |
| #2 | #1 AND #2 | 65 (2 protocols, 6 reviews and 57 trials) |
| #2 | Search dexmedetomidine* OR Precedex* OR DEX | 4938 |
| #1 | Search magnesium* | 7034 |

CNKI

#1 magnesium*

#2 dexmedetomidine*

#3 #1 AND #2 (8)

| Search | Query | Items found |
| --- | --- | --- |
| #3 | #1 AND #2 | 8 |
| #2 | Search " dexmedetomidine*" in Chinese |  |
| #1 | Search " magnesium*" in Chinese |  |
